# Long-term anxiety of natural and biological hazards on community and healthcare workers

**DOI:** 10.3389/fpsyt.2025.1702488

**Published:** 2025-11-14

**Authors:** Po-Fei Chen, Hsuan Lung, Mei-Chung Chang, For-Wey Lung

**Affiliations:** 1 Calo Psychiatric Center, Pingtung County, Taiwan; 2 Department of Dentistry, Kaohsiung Chang Gung Memorial Hospital, Kaohsiung, Taiwan; 3 Department of Dentistry, Kaohsiung Municipal Fong Shan Hospital - Under the Management of Chang Gung Medical Foundation, Kaohsiung, Taiwan; 4 Department of Dentistry, Kaohsiung Municipal Ta-Tung Hospital, Kaohsiung, Taiwan; 5 School of Dentistry and Institute of Oral Medicine, College of Medicine, National Cheng Kung University, Tainan, Taiwan; 6 School of Medicine for International Students, College of Medicine, I-Shou University, Kaohsiung, Taiwan; 7 Graduate Institute of Medical Science, National Defense Medical University, Taipei, Taiwan; 8 International Graduate Program of Education and Human Development, National Sun Yat-Sen University, Kaohsiung, Taiwan; 9 Institute of Education, National Sun Yat-Sen University, Kaohsiung, Taiwan

**Keywords:** natural disasters, typhoon, SARS, COVID-19, healthcare workers, mental distress

## Abstract

**Background:**

Exposure to natural and biological hazards has been linked to long-term declines in mental health. However, limited research has examined the sustained psychological impact of these disasters over extended follow-up periods.

**Methods:**

This study investigated the long-term mental health consequences of natural and biological hazards among healthcare workers and community residents through three longitudinal datasets collected over two decades. Data sources included: (1) 127 healthcare workers exposed to Severe Acute Respiratory Syndrome (SARS) in 2003, with 123 followed up one year later; (2) 152 community residents affected by Typhoon Morakot in 2009, with 125 followed up 1.5 years later; and (3) 458 healthcare workers affected by Coronavirus disease 2019 (COVID-19) in 2020, with 321 followed up two years later.

**Results:**

Findings show that mental distress prevalence among community residents remained stable (1.6% initially vs. 1.5% at follow-up), whereas healthcare workers exhibited increasing distress over time (SARS: 4.7% to 15.4%; COVID-19: 9.7% to 11.8%). Pathway modeling revealed that initial anxiety at the onset of SARS, Typhoon Morakot, and COVID-19 was a strong predictor of long-term psychological distress.

**Conclusions:**

These results highlight the importance of sustained mental health interventions for healthcare workers facing prolonged exposure to stressors during biological disasters. In addition to early anxiety screening, system-level measures such as adequate staffing, transparent communication, and institutional preparedness are essential to mitigate long-term psychological consequences.

## 1 Introduction

Biological and natural hazards can both trigger fear for personal well-being, uncertainty about the future, abrupt disruptions to daily life, and resource limitations (1). Both types of hazards can be classified as potentially traumatic events, as they may involve actual or threatened death, serious injury, or violence, as defined by the *Diagnostic and Statistical Manual of Mental Disorders, 5th Edition, Text Revision* (DSM-5-TR). Exposure to disaster-related stressors is associated with declines in mental health, including depression, post-traumatic stress disorder (PTSD), fear, suicidal behavior, and other psychiatric symptoms such as mood disturbances and loss of interest in activities (2). Psychological and physical responses to trauma vary based on event characteristics, social context, and an individual’s past experiences and expectations (3). Mental health outcomes following disasters are often linked to destruction and changes in the physical environment (4).

The Coronavirus disease 2019 (COVID-19) pandemic significantly increased stress levels due to uncertainties, fear of infection, and lockdown measures, all of which radically altered daily life and negatively impacted mental health. Individuals reported heightened levels of stress, anxiety, depressive symptoms, insomnia, denial, anger, and fear (5). Healthcare workers, being on the front lines of patient care, were particularly vulnerable to both infection and psychological distress (6). Meta-analyses and systematic reviews consistently indicate high rates of anxiety, depression, and insomnia among healthcare workers during the pandemic (7). Furthermore, a longitudinal study in China found that community residents experienced persistent peritraumatic stress, anxiety, and depression, with no significant improvement over time (8).

A similar pattern was observed during the Severe Acute Respiratory Syndrome (SARS) outbreak, which was first identified in China on November 16, 2002. The outbreak ultimately infected 8,096 individuals across 29 countries, leading to 774 deaths (9). In Taiwan, 346 people were infected, including 105 healthcare workers. Of the 37 fatalities (21% mortality rate), seven were healthcare professionals. Due to the heightened risk of infection, psychiatric morbidity among hospital staff reached 75% during the SARS outbreak (10), reflecting the immense psychological burden experienced by frontline healthcare workers (11).

Like pandemics, natural hazards can cause significant mental distress due to property damage, physical injuries, prolonged disruption of daily life, and displacement of individuals and families (12). On August 7, 2009, Typhoon Morakot, a Category 2 typhoon, struck Taiwan, bringing 2,500 mm of rainfall over three days. The heavy precipitation triggered landslides that destroyed buildings and entire villages in mountainous regions of southern Taiwan, resulting in 681 deaths and 18 missing persons. Large-scale natural disasters of this magnitude are associated with increased prevalence of psychiatric disorders, PTSD, and anxiety (13). Studies found that 2.4% of affected community residents reported psychological distress within one month of the disaster, increasing to 4.0% one year later (14).

Although both biological disasters (e.g., SARS and COVID-19) and natural hazards (e.g., Typhoon Morakot) are classified as traumatic events that adversely affect mental health, they may elicit distinct patterns of psychological symptoms and varying degrees of long-term distress. This study aimed to investigate the long-term psychological impact of biological disasters on healthcare workers and natural disasters on community residents in southern Taiwan.

Specifically, the study sought to:

1. Examine the severity and persistence of specific psychological symptoms, including anxiety, depression, hostility, interpersonal sensitivity/inferiority, and insomnia, assessed using the five-item Brief Symptom Rating Scale (BSRS-5) among healthcare workers following the SARS outbreak (one-year follow-up) and the COVID-19 pandemic (two-year follow-up).
2. Assess the same set of psychological symptoms among community residents affected by Typhoon Morakot, both immediately after the disaster and at 1.5-year follow-up.
3. Investigate the predictive role of these individual psychological symptoms in determining long-term psychological distress, as measured by the BSRS-5.

By delineating symptom-specific trajectories across disaster types and timeframes, this study aims to clarify how different forms of traumatic exposure shape psychological outcomes and to inform the development of targeted interventions at both individual and systemic levels.

## 2 Materials and methods

### 2.1 Participants

#### 2.1.1 SARS (2003–2004)

Healthcare workers were recruited from a general hospital in Southern Taiwan during the SARS outbreak (July 2003–March 2004). A total of 127 healthcare workers initially agreed to participate, and 123 (96.6%) completed the follow-up assessment one year later (15).

#### 2.1.2 Typhoon Morakot (2009–2011)

Community residents from Chia-Tung, Pingtung County—one of the most severely affected areas—were recruited one month after Typhoon Morakot (September 2009). The disaster caused severe flooding, reaching up to two stories high. A total of 152 participants were enrolled at baseline, with 125 (82.2%) completing follow-up 1.5 years later (March–August 2011) (14).

#### 2.1.3 COVID-19 healthcare workers (2020–2022)

Healthcare workers were recruited from three hospitals in Southern Taiwan (two general hospitals and one psychiatric hospital) using convenience sampling. At baseline (February 2020), 458 healthcare workers participated: 276 from the first general hospital, 98 from the second general hospital, and 84 from the psychiatric hospital. At the two-year follow-up (April–July 2022), 321 (70.1%) participants remained: 213 from the first general hospital, 57 from the second general hospital, and 51 from the psychiatric hospital (16).

This study adhered to the ethical standards of the 1964 Declaration of Helsinki and its later amendments. Ethical approval was granted by the Institutional Review Board of Kaohsiung Armed Forces General Hospital (Approval Number: KAFGH 112-011). All data were fully anonymized before analysis. Since this study involved secondary data analysis, the requirement for informed consent was waived by the Institutional Review Board.

### 2.2 Measures

#### 2.2.1 Mental health assessment

All participants completed a demographic questionnaire and a mental health assessment at both baseline and follow-up. The five-item Brief Symptom Rating Scale (BSRS-5) was used in all groups except for SARS participants, who completed the Chinese Health Questionnaire (CHQ) at follow-up.

#### 2.2.2 Brief-Symptom Rating Scale - 5

The BSRS-5 is a validated mental health screening tool assessing five symptom domains: Anxiety, depression, hostility, interpersonal sensitivity/inferiority, and insomnia. The Chinese version of the BSRS-5 is widely used in Taiwan to screen mental health conditions in psychiatric inpatients, general medical patients, community residents, and healthcare professionals (16, 17). A cutoff score of ≥10 indicates mental distress in healthcare workers (16). This scale was used to assess mental health among healthcare workers during the SARS and COVID-19 pandemics and community residents following Typhoon Morakot.

#### 2.2.3 Chinese Health Questionnaire

The CHQ-12 is a self-reported mental health screening instrument derived from the General Health Questionnaire (GHQ) (18). It was specifically developed to assess psychiatric morbidity in Chinese-speaking populations (18). The CHQ-12 evaluates three mental health dimensions: Somatic symptoms, anxiety, depression. A cutoff score of ≥3 indicates the presence of psychiatric symptoms (11, 15). The CHQ-12 was used to assess the mental health of healthcare workers following the SARS outbreak.

### 2.3 Statistical analysis

Descriptive statistics were first computed to summarize the demographic characteristics of healthcare workers exposed to SARS and COVID-19, as well as community residents affected by Typhoon Morakot. To compare BSRS-5 total and individual symptom scores between baseline and follow-up assessments, paired t tests were performed.

Repeated-measures analyses of variance (ANOVA) were then conducted to examine within-subject changes in psychological symptoms across two time points (baseline and follow-up) and to determine whether these temporal changes differed by demographic factors, including sex and marital status. Bonferroni-adjusted pairwise comparisons were applied to control for Type I error in *post hoc* analyses as corrections for multiple comparisons.

To assess the association between initial psychological symptoms and long-term mental distress, generalized estimating equations (GEE) were employed. GEE is well-suited for analyzing repeated measures data and was used to determine which BSRS-5 symptoms at baseline were predictive of mental distress at one-year (SARS), 1.5-year (Typhoon Morakot), or two-year (COVID-19) follow-up.

To further explore predictive relationships among BSRS-5 symptoms, structural equation modeling (SEM) was conducted. SEM was used to construct and validate a factor analysis pathway model, examining which BSRS-5 symptoms at baseline were associated with psychological symptoms at follow-up. Model fit was evaluated using the χ² goodness-of-fit test, where a non-significant χ² value (p > 0.05) indicated a good fit. Additional model fit indices included: Adjusted goodness-of-fit index (AGFI) > 0.90, root mean square error of approximation (RMSEA) < 0.05. Only parsimonious SEM models were presented, meaning that only statistically significant pathways (p < 0.05) were retained.

All descriptive and GEE analyses were conducted using Statistical Package for the Social Sciences (SPSS) 26.0 for Windows (SPSS Inc., Chicago, USA). SEM analyses were performed using the Analysis of a MOment Structures 26.0 (SPSS Inc., Chicago, USA).

## 3 Results

### 3.1 Demographic characteristics and symptom comparisons

The demographic distribution of SARS healthcare workers, Typhoon Morakot community residents, and COVID-19 healthcare workers is presented in **Table 1**. Paired t-tests showed statistically significant differences in BSRS-5 total scores and symptom scores (anxiety, depression, hostility, inferiority, and insomnia) between baseline and follow-up assessments for participants affected by Typhoon Morakot and COVID-19 (**Table 1**).

**Table 1:** Demographic characteristics and the five-item Brief-Symptom Rating Scale (BSRS-5) comparison during the disaster and follow-up.

	SARS (N = 123)		Typhoon morakot (N = 125)			COVID-19 (N = 321)		
Variable	N (%)		N (%)			N (%)		
Male Female Married	50 (39.4) 73 (60.6) 73 (59.3)		56 (44.8) 69(55.2) 92 (73.6)			25 (7.8) 296(92.2) 196 (61.1)		
	Mean (SD)		Mean (SD)			Mean (SD)		
Age	32.54 (6.91)		40.29 (11.16)			40.09 (9.51)		
	Amidst disaster	1-year Follow-up	Amidst disaster	1.5-year Follow-up		Amidst disaster	2-years Follow-up	
	n (%)	n (%)	n (%)	n (%)		n (%)	n (%)	
BSRS-5≧10	6 (4.7)	CHQ≧3 19 (15.4)	2 (1.6)	2 (1.6)		31 (9.7)	38 (11.8)	
	Mean (SD)	Mean (SD)	Mean (SD)	Mean (SD)	*t*	Mean (SD)	Mean (SD)	*t*
BSRS-5 total score	3.74 (3.50)	CHQ 1.27 (2.14)	2.23 (3.19)	1.80 (2.94)	7.83**	4.14 (3.68)	4.68 (3.75)	20.17**
Anxiety	0.73 (0.80)	0.32 (0.58)	0.45 (0.67)	0.42 (0.71)	7.53**	0.84 (0.82)	0.91 (0.85)	18.33**
Depression	0.88 (0.82)	0.24 (0.59)	0.55 (0.82)	0.42 (0.85)	7.55**	0.75 (0.92)	0.85 (0.85)	14.64**
Hostility	0.84 (0.92)	Somatic 0.29 (0.72)	0.50 (0.79)	0.27 (0.60)	7.14**	1.01 (0.95)	1.13 (0.93)	19.05**
Inferiority	0.59 (0.85)		0.19 (0.62)	0.21 (0.56)	3.47**	0.59 (0.83)	0.69 (0.80)	12.80**
Insomnia	0.69 (1.00)		0.54 (0.80)	0.48 (0.82)	7.50**	0.95 (1.00)	1.06 (1.01)	17.05**

91. *p<.05; **p<.01

Repeated-measures ANOVA for symptom scores (anxiety, depression, hostility, inferiority, and insomnia) by time, sex and marital status was further analyzed in Typhoon Morakot and COVID-19 datasets, to determine whether these changes differed across demographic groups. In the Typhoon dataset, a significant interaction between sex and time was found for anxiety symptoms, F(1, 123) = 5.10, p = .026, indicating that the pattern of change in anxiety over time differed between males and females. Specifically, females showed a significant difference in anxiety levels across time, whereas males did not. Significant interactions were also observed between marital status and time for anger and inferiority, F(1, 123) = 8.85, p = .004, and F(1, 123) = 6.35, p = .013, respectively, suggesting that changes in anger and inferiority across time varied according to marital status. Married participants exhibited more pronounced changes in anger and inferiority compared to unmarried participants.

In the COVID-19 dataset, no significant interactions between sex and time were observed for any of the five psychological symptoms. However, significant interactions were found between marital status and time for anger and insomnia, F(1, 123) = 6.08, p = .014, and F(1, 123) = 15.94, p <.001, respectively, indicating that changes in these symptoms across time varied by marital status. Specifically, married participants exhibited more pronounced changes in anger and insomnia over time compared to unmarried participants.

### 3.2 Generalized estimating equations analysis

GEE analysis was used to identify BSRS-5 symptoms at baseline that were associated with mental distress at follow-up (BSRS-5 ≥10).

As shown in **Table 2**, individuals who reported higher anxiety levels during the typhoon were more likely to experience mental distress 1.5 years later (β = 2.68, p < 0.001). Conversely, individuals with lower depression levels at baseline were also more likely to develop mental distress at follow-up (β = -0.84, p = 0.015).

**Table 2:** Generalized equation estimation results of the Brief-Symptom Rating Scale (BSRS-5) symptoms amidst the disaster associated with mental distress at follow-up.

Dependent variable	Independent variable	ß	S.e.	95% C.I.	P
Typhoon BSRS-5≧10 at follow-up	Time	0.02	0.511	–0.98 to 01.03	0.963
	Anxiety	2.68	0.290	2.11 to 3.25	<0.001
	Hostility	0.79	0.634	–0.45 to 2.03	0.212
	Depression	–0.84	0.023	–1.52 to –0.16	0.015
	Inferiority	–0.58	0.449	–1.46 to 0.30	0.194
	Insomnia	–0.79	0.195	–0.08 to 0.03	0.116
COVID-19 BSRS-5≧10 at follow-up	Time	–0.01	0.007	–0.03 to 0.004	0.167
	Anxiety	0.44	0.133	0.02 to 0.07	0.001
	Hostility	0.02	0.011	-0.01 to 0.04	0.130
	Depression	0.09	0.014	0.06 to 0.11	<0.001
	Inferiority	0.11	0.015	0.08 to 0.14	<0.001
	Insomnia	0.06	0.010	0.04 to 0.07	<0.001

GEE analysis also examined which BSRS-5 symptoms during the COVID-19 pandemic predicted mental distress at two-year follow-up. Results indicated that healthcare workers who reported higher levels of anxiety, depression, inferiority, and insomnia during the pandemic had significantly increased odds of experiencing mental distress at follow-up (β = 0.44, p = 0.001; β = 0.09, p < 0.001; β = 0.11, p < 0.001; β = 0.06, p < 0.001).

### 3.3 Structural equation modeling analysis

SEM was used to examine pathway relationships between BSRS-5 symptoms at baseline and follow-up for participants exposed to SARS, Typhoon Morakot, and COVID-19.

**Figure 1:**
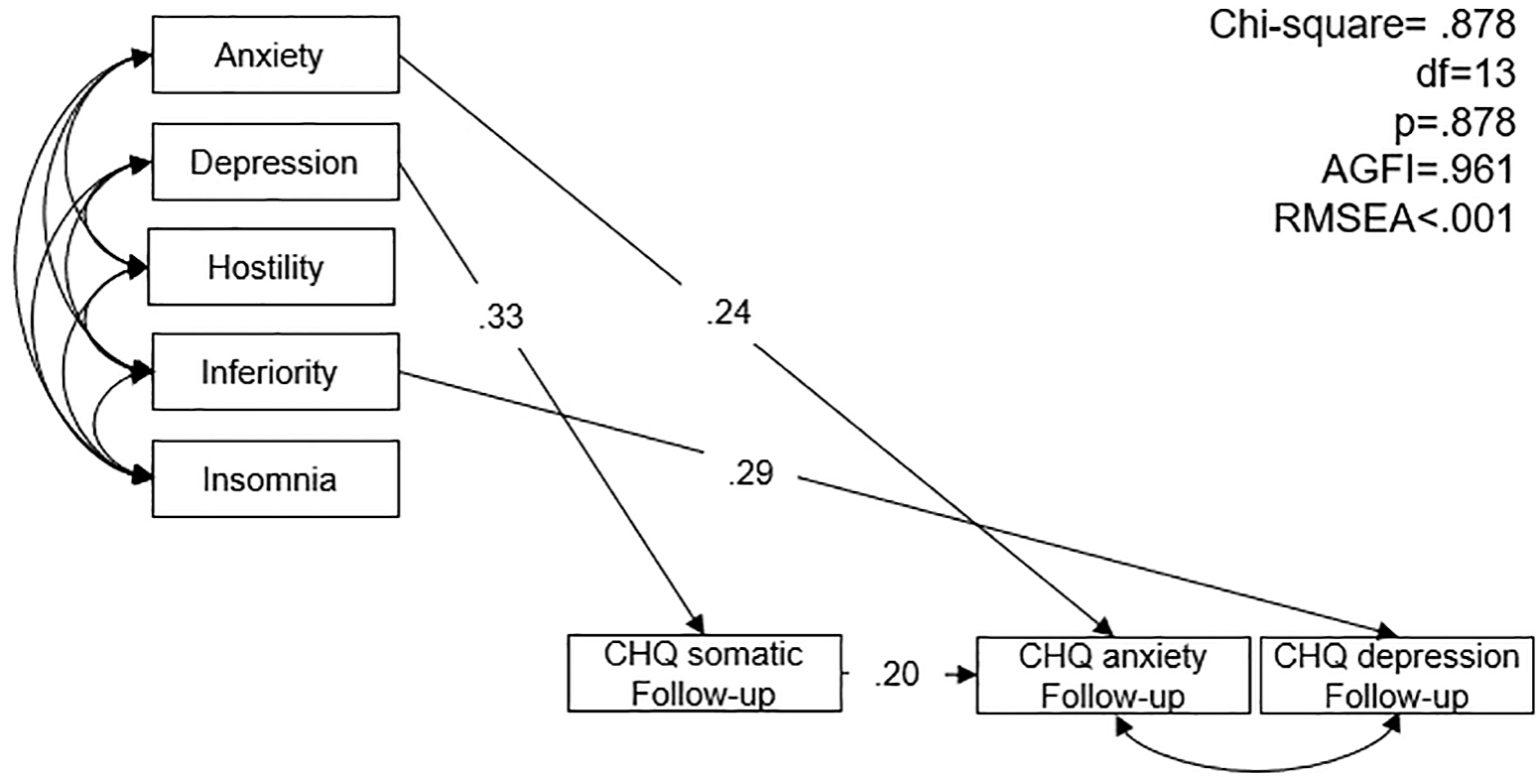
Structural equation model of Brief-Symptom Rating Scale symptom of the healthcare workers during SARS and the Chinese health questionnaire symptoms at one-year follow-up. AGFI: adjusted goodness-of-fit; RMSEA: root mean square error of approximation.

The first SEM model assessed associations between BSRS-5 symptoms during SARS and Chinese Health Questionnaire (CHQ) symptoms at one-year follow-up. The model resulted in a *p* of 0.878 (>.05), AGFI of 0.961 (> 0.9), and RMSEA of < 0.001 (< 0.08) implied that the null model approximates the real structure, as shown in **Figure 1**. Higher baseline anxiety was associated with higher CHQ anxiety at follow-up (β = 0.24, p = 0.002). Higher baseline depression was associated with increased somatic symptoms (β = 0.33, p < 0.001). Higher inferiority scores were associated with higher depression at follow-up (β = 0.29, p < 0.001). Somatic symptoms at follow-up were positively associated with anxiety symptoms (β = 0.20, p = 0.013).

**Figure 2:**
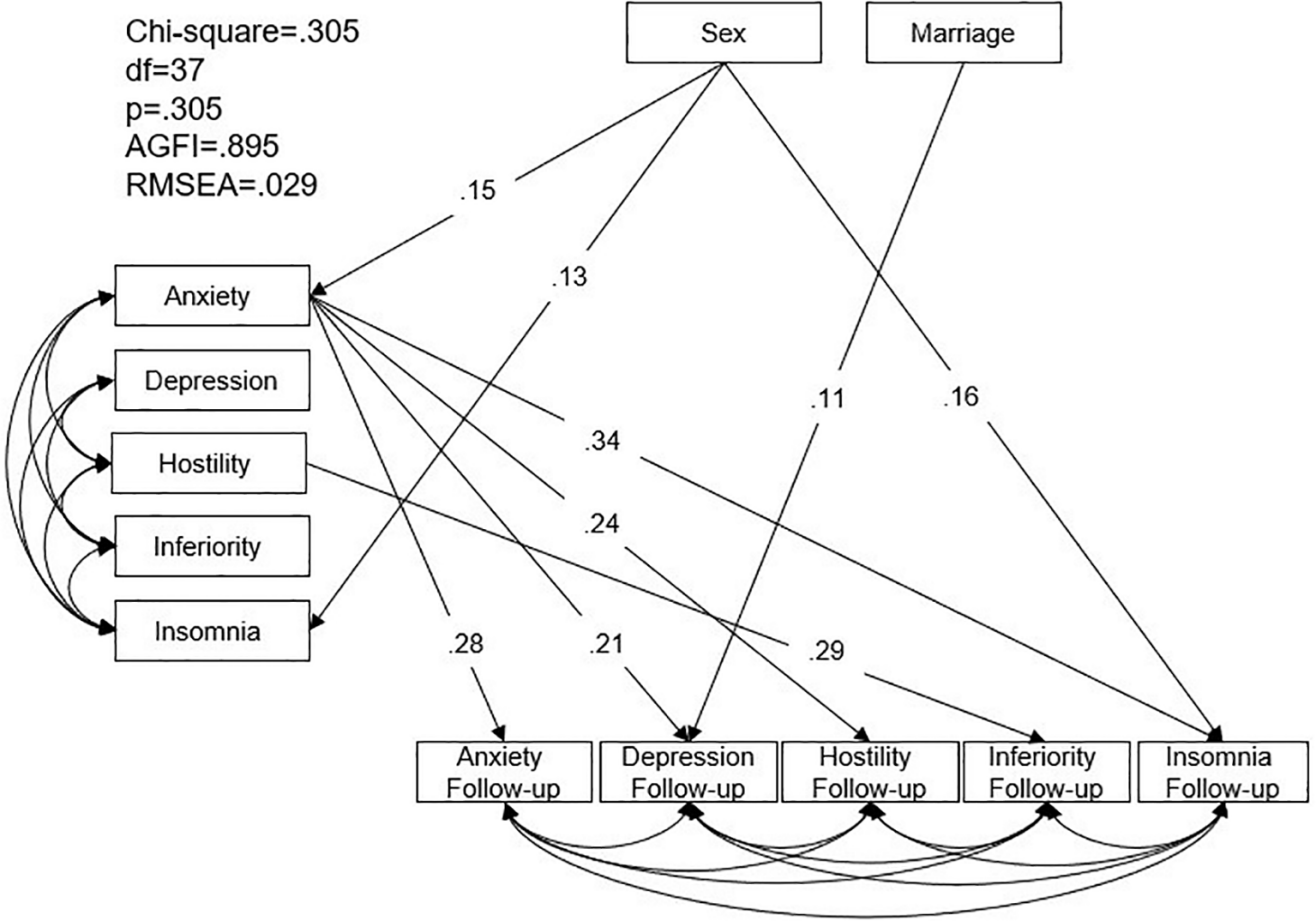
Structural equation model of Brief-Symptom Rating Scale symptom of the community residents during Typhoon Morakot and at one-year follow-up. AGFI: adjusted goodness-of-fit; RMSEA: root mean square error of approximation.

The second SEM model examined relationships between BSRS-5 symptoms during Typhoon Morakot and symptoms at 1.5-year follow-up. The model demonstrated good fit (χ² = 0.305, p > 0.05; AGFI = 0.895; RMSEA = 0.029) (**Figure 2**). Female participants reported higher anxiety and insomnia at baseline and higher insomnia at follow-up (β = 0.15, p = 0.014; β = 0.13, p = 0.036; β = 0.16, p = 0.008). Married participants had higher depression levels at follow-up (β = 0.11, p = 0.025). Higher baseline anxiety was associated with higher levels of anxiety, depression, hostility, and insomnia at follow-up (β = 0.28, p < 0.001; β = 0.21, p < 0.001; β = 0.24, p = 0.004; β = 0.34, p < 0.001). Higher hostility levels at baseline predicted higher inferiority levels at follow-up (β = 0.38, p < 0.001).

**Figure 3:**
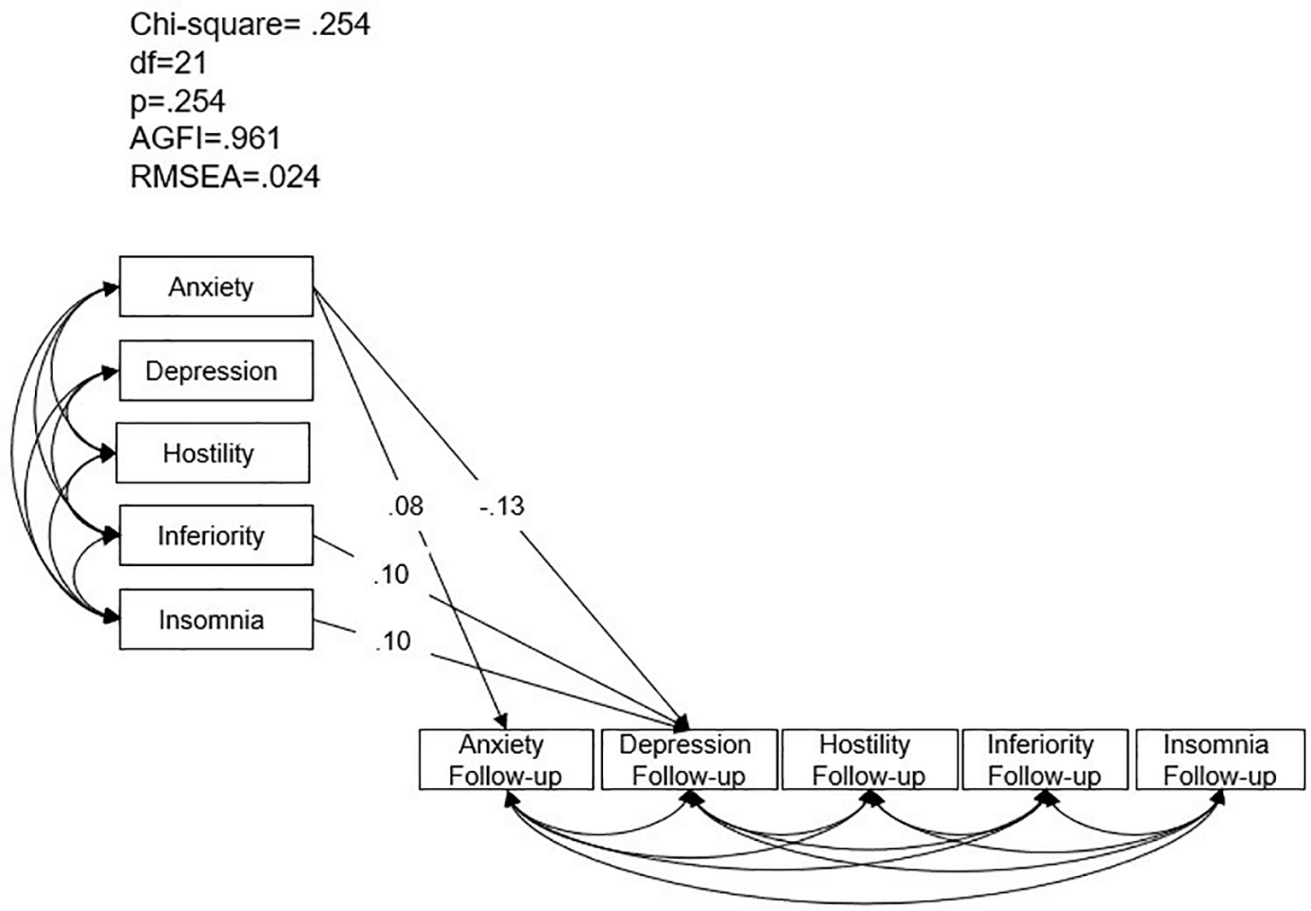
Structural equation model analysis of Brief-Symptom Rating Scale-5 symptoms in healthcare workers during the COVID-19 pandemic and at two-year follow-up. AGFI: adjusted goodness-of-fit; RMSEA: root mean square error of approximation.

The third SEM model investigated associations between BSRS-5 symptoms at the onset of COVID-19 and mental health symptoms at two-year follow-up. The model demonstrated good fit (χ² = 0.254, p > 0.05; AGFI = 0.961; RMSEA = 0.024) (**Figure 3**). Higher baseline anxiety was associated with higher anxiety and lower depression at follow-up (β = 0.08, p = 0.015; β = -0.13, p = 0.036). Higher baseline inferiority and insomnia were associated with higher depression levels at follow-up (β = 0.10, p = 0.021; β = 0.10, p = 0.030).

## 4 Discussion

This study examined the long-term psychological impact of natural and biological disasters on community residents and healthcare workers. Our findings indicate that the mental distress patterns differ between these two groups, with community residents maintaining stable mental health over time, while healthcare workers experienced escalating distress in prolonged exposure to biological disasters. For natural hazards, the prevalence of mental distress among community residents affected by Typhoon Morakot remained stable (1.6%) at both baseline and 1.5-year follow-up. In contrast, for biological disasters, mental distress among healthcare workers significantly increased over time. During the SARS outbreak, 4.7% of healthcare workers reported distress, which increased to 15.4% at follow-up. Similarly, during the COVID-19 pandemic, distress levels rose from 9.7% to 11.8% over two years. Additionally, BSRS-5 total scores and symptom scores (anxiety, depression, hostility, inferiority, and insomnia) showed significant increases at follow-up, particularly among healthcare workers. Both GEE and SEM analyses consistently showed that higher anxiety levels at the onset of SARS, Typhoon Morakot, and COVID-19 were predictive of increased mental distress at follow-up, suggesting that initial anxiety may serve as a screening indicator for long-term psychological outcomes.

A comparison of Typhoon Morakot (natural hazard) with SARS and COVID-19 (biological disasters) revealed that mental distress in community residents did not escalate over time, while healthcare workers under prolonged exposure to SARS and COVID-19 experienced worsening psychological distress. This distinction aligns with the concept of continuous traumatic stress (Type III trauma), which is more severe than single-event trauma (Type I) or recurrent traumatic episodes (Type II) (19). Unlike natural hazards, where the trauma is time-limited, biological disasters impose prolonged exposure to stressors, leading to sustained psychological strain and vulnerability beyond the tolerated threshold (20). Healthcare workers face persistent stressors, including infection risks, prolonged uncertainty, workload pressure, social stigma, and moral injury (21). The COVID-19 pandemic, in particular, presented a multilayered trauma, with psychological fears (infection, mortality), social disruptions (lockdowns, isolation), and economic hardships (job loss, financial instability) (20). These factors contribute to higher mental distress at long-term follow-up.

The increased mental distress among healthcare workers at follow-up is concerning. Similar findings were reported following SARS, where anxiety, depression, and post-traumatic stress levels remained elevated one year after the outbreak (22). Systematic reviews confirm that COVID-19 healthcare workers exhibited high levels of anxiety, depression, and insomnia (22). Factors such as fear of infection, concerns about family transmission, workload strain, and social stigma place healthcare workers at heightened psychological risk (23, 24). Notably, many healthcare workers may suppress emotional distress during the peak of a crisis due to professional expectations (25). As the immediate threat subsides, denial mechanisms weaken, leading to a “rebound effect” in emotional responses (22). This explains the increased distress at follow-up despite the resolution of the crisis.

Pathway analysis further revealed that SARS healthcare workers who experienced higher depression at baseline were more likely to report somatic symptoms at follow-up. This aligns with research showing that healthcare workers, particularly in collectivist cultures, may suppress emotions and exhibit alexithymic traits due to professional and cultural expectations (17, 26). In Chinese culture, emotional restraint and social harmony are highly valued, leading individuals to express psychological distress through somatic symptoms rather than overt emotional expression (26). In contrast to healthcare workers, community residents exhibited better mental health outcomes at follow-up than immediately post-typhoon. While initial distress levels were elevated, they gradually declined over time. This aligns with prior studies indicating that, despite experiencing post-traumatic stress symptoms, depression, sleep disturbances, and anxiety, most individuals do not develop long-term psychopathology and eventually return to baseline functioning (27). GEE results showed that anxiety, depression, hostility, and insomnia levels were significantly higher immediately after Typhoon Morakot but decreased at 1.5-year follow-up. SEM analysis further identified that higher hostility immediately post-typhoon predicted greater feelings of inferiority at follow-up. Anger has been recognized as a key factor in post-traumatic stress responses, often serving as a defensive mechanism against deeper emotions like fear, anxiety, or loss (28). The DSM-5-TR highlights that anger and aggression are common features of trauma- and stress-related disorders, particularly when individuals perceive failures in disaster response or inadequate protection from authorities (29). A key takeaway from this study is that anxiety at the onset of a disaster is a significant predictor of long-term psychological distress, regardless of whether the event is a natural hazard or a biological disaster. Early identification of high-anxiety individuals may allow for targeted interventions to mitigate long-term mental health risks.

This study has some limitations. CHQ was only collected at the one-year follow-up for SARS healthcare workers, preventing a direct comparison of their initial and follow-up mental health status. However, both CHQ and BSRS-5 are validated mental health screening tools and widely used in Chinese populations (16, 17, 30). Another limitation is that community residents were analyzed for Typhoon Morakot, while healthcare workers were analyzed for SARS and COVID-19, leading to potential differences in psychological responses based on occupational roles. Additionally, demographic variables were recorded in simplified format of binary variables for sex and marital status. As a result, the current analyses could not account for these additional demographic nuances, which represents a limitation in interpreting subgroup differences in psychological responses. However, despite these differences, all three datasets consistently showed that initial anxiety levels predicted long-term mental distress, suggesting that anxiety-based screening is applicable across different populations.

A major strength of this study is its longitudinal design spanning nearly 20 years, encompassing the 2003 SARS outbreak, 2009 Typhoon Morakot, and 2020 COVID-19 pandemic. The study’s robust follow-up periods (one, 1.5, and two years) provide valuable insights into the long-term psychological impact of disasters. Despite differences in cohorts, disaster types, and assessment tools, the consistent finding that initial anxiety predicts long-term distress underscores its clinical relevance.

This study highlights the differential psychological impacts of natural and biological disasters. While community residents affected by a natural hazard exhibited resilience over time, healthcare workers exposed to biological disasters experienced worsening distress due to prolonged exposure and continuous traumatic stress. Across all datasets, higher initial anxiety consistently predicted greater psychological distress at follow-up, suggesting that anxiety screening at disaster onset may help identify at-risk individuals for early intervention. From a public health perspective, stakeholders, clinicians, and policymakers should prioritize both individual and systemic approaches to mental health support, particularly for high-risk groups like healthcare workers. In addition to implementing culturally relevant anxiety relief techniques, such as mindfulness or religious coping strategies (16), system-level preventive measures are crucial. These include ensuring adequate staffing and rest periods, providing accurate and transparent information during outbreaks, strengthening institutional preparedness, and fostering supportive workplace cultures that reduce chronic stress exposure. Such comprehensive strategies may be more effective in preventing long-term psychological distress and promoting workforce resilience. Future research should explore how these systemic interventions interact with individual-level factors and examine longer-term psychological trajectories beyond two years.

## Data Availability

The raw data supporting the conclusions of this article will be made available by the authors, without undue reservation.
